# Supplementary material for: Novel organ-specific genetic factors for quantitative resistance to late blight in potato
Source: PLoS One. 2019 Jul 16;14(7):e0213818. doi: 10.1371/journal.pone.0213818 (PMC6634379; doi:10.1371/journal.pone.0213818)
Supplement: S2 Table — a. Analysis of variance for genotype, environment and genotype x environment among the association panel for a. leaves phenotyping. b. stem phenotyping (p<0.001). (PDF) [file pone.0213818.s002.pdf]

S2 Table. Analysis of variance. a. Analysis of variance for genotype, environment and genotype x environment among the association panel for a. leaves phenotyping. b. stem phenotyping (p<0.001).

**a.**	**DF**	**SS**	**MS**	**F**	**Prob. F**
**ENV**	2	126.086	0.63043	16.710.523	0
**GEN**	104	836.923	0.08047	2.133.073	0
**ENV*GEN**	208	315.455	0.01517	402.002	0
**Residuals**	630	237.677	0.00377		
**b.**					
**ENV**	2	0.27535	0.13767	470.447	0
**GEN**	104	331.535	0.03188	1.089.325	0
**ENV*GEN**	208	149.348	0.00718	245.356	0
**Residuals**	630	184.365	0.00293		

The historical data were not included in the analysis.
