# Supplementary material for: Novel organ-specific genetic factors for quantitative resistance to late blight in potato
Source: PLoS One. 2019 Jul 16;14(7):e0213818. doi: 10.1371/journal.pone.0213818 (PMC6634379; doi:10.1371/journal.pone.0213818)
Supplement: S4 Table — Transitions and transversion SNPs from the genotyping matrix of 83,862 SNPs. (PDF) [file pone.0213818.s004.pdf]

S4 Table. Transitions and transversion data. Transitions and transversion SNPs from the genotyping matrix of 83,862 SNPs.

	SNP	# Markers	Total SNP
**Transitions**	A/G - G/A	24,986	49,948
	C/T - T/C	24,962	
**Trasversions**	A/C - C/A	8,551	33,914
	G/T - T/G	8,548	
	A/T - T/A	11,21	
	C/G - G/C	5,605	
