# Supplementary material for: Novel organ-specific genetic factors for quantitative resistance to late blight in potato
Source: PLoS One. 2019 Jul 16;14(7):e0213818. doi: 10.1371/journal.pone.0213818 (PMC6634379; doi:10.1371/journal.pone.0213818)
Supplement: S1 Fig — a. GBS and b. 2b-RAD. K indicate the specific number of population generated by the STRUCTURE software. (PDF) [file pone.0213818.s006.pdf]

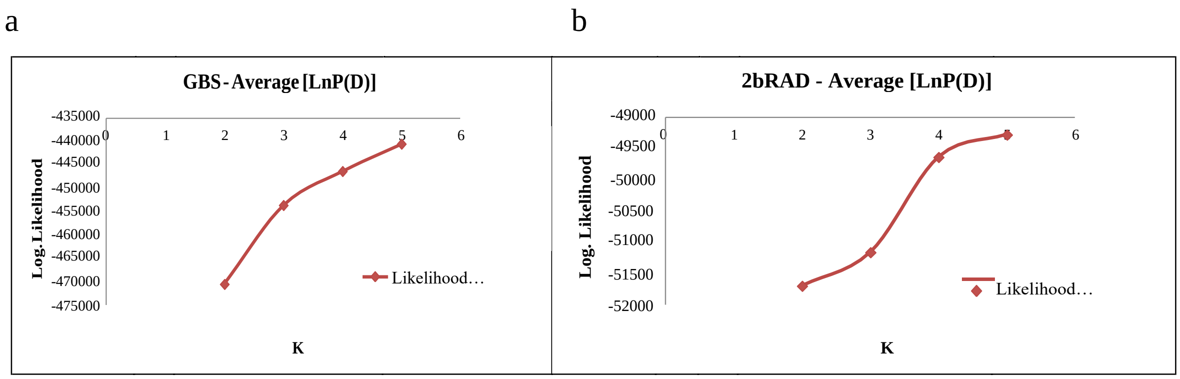

S1 Fig. Likelihood of population assignment to Group Phureja genotypes employing two genotyping strategies. a. GBS and b. 2b-RAD. K indicate the specific number of population generated by the STRUCTURE software
