# Supplementary material for: Novel organ-specific genetic factors for quantitative resistance to late blight in potato
Source: PLoS One. 2019 Jul 16;14(7):e0213818. doi: 10.1371/journal.pone.0213818 (PMC6634379; doi:10.1371/journal.pone.0213818)
Supplement: S2 Fig — Kinship estimated using the efficient massive mapping algorithm (EMMA) based on 4,216 SNP-GBS from Group Phureja genotypes. (PDF) [file pone.0213818.s007.pdf]

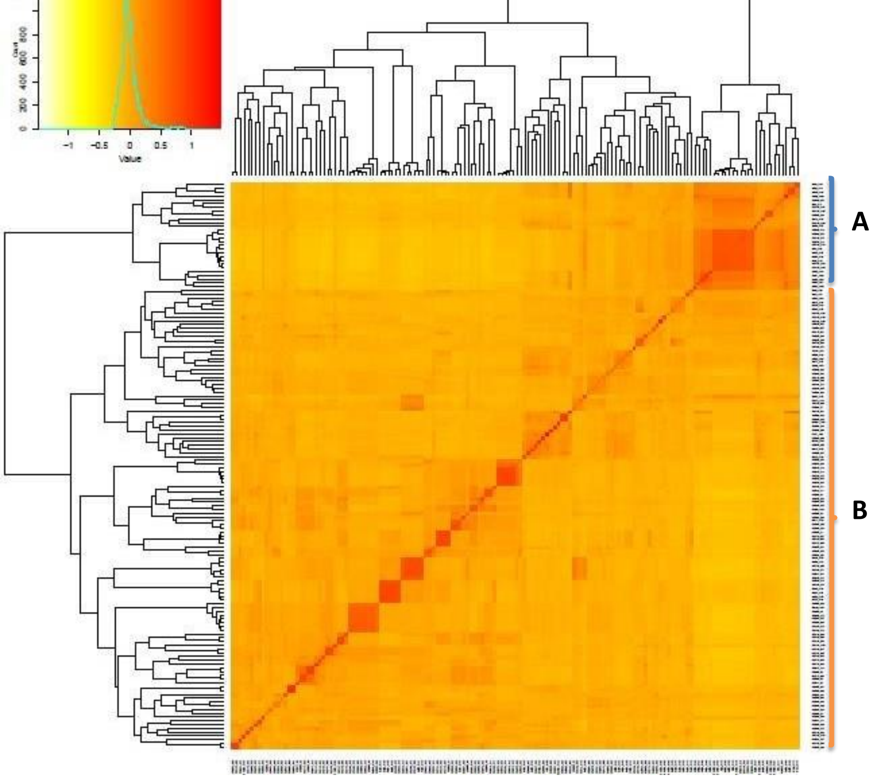

S2 Fig. Heatmap and dendrogram of a kinship matrix. Kinship estimated using the efficient massive mapping algorithm (EMMA) based on 4,216 SNP-GBS from Group Phureja genotypes.
