# Supplementary material for: Novel organ-specific genetic factors for quantitative resistance to late blight in potato
Source: PLoS One. 2019 Jul 16;14(7):e0213818. doi: 10.1371/journal.pone.0213818 (PMC6634379; doi:10.1371/journal.pone.0213818)
Supplement: S3 Fig — On the x-axis, different color tones correspond to different chromosomes within the genome from 1 to 12. The dashed horizontal line indicates the significant threshold at-log 10 (p- value) = 8. a.- d. Manhattan plot and putative QTL for resistance to P.infestans in foliage. a-b Correspond to SNP associated in La Union environment, c-d Correspond to SNP associated in Subachoque environment. e.-h. Manhattan plot for resistance to P.infestans in stem. e-f Correspond to SNP associated in La Union environment, g-h. Correspond to SNP associated in Subachoque environment. (PDF) [file pone.0213818.s008.pdf]

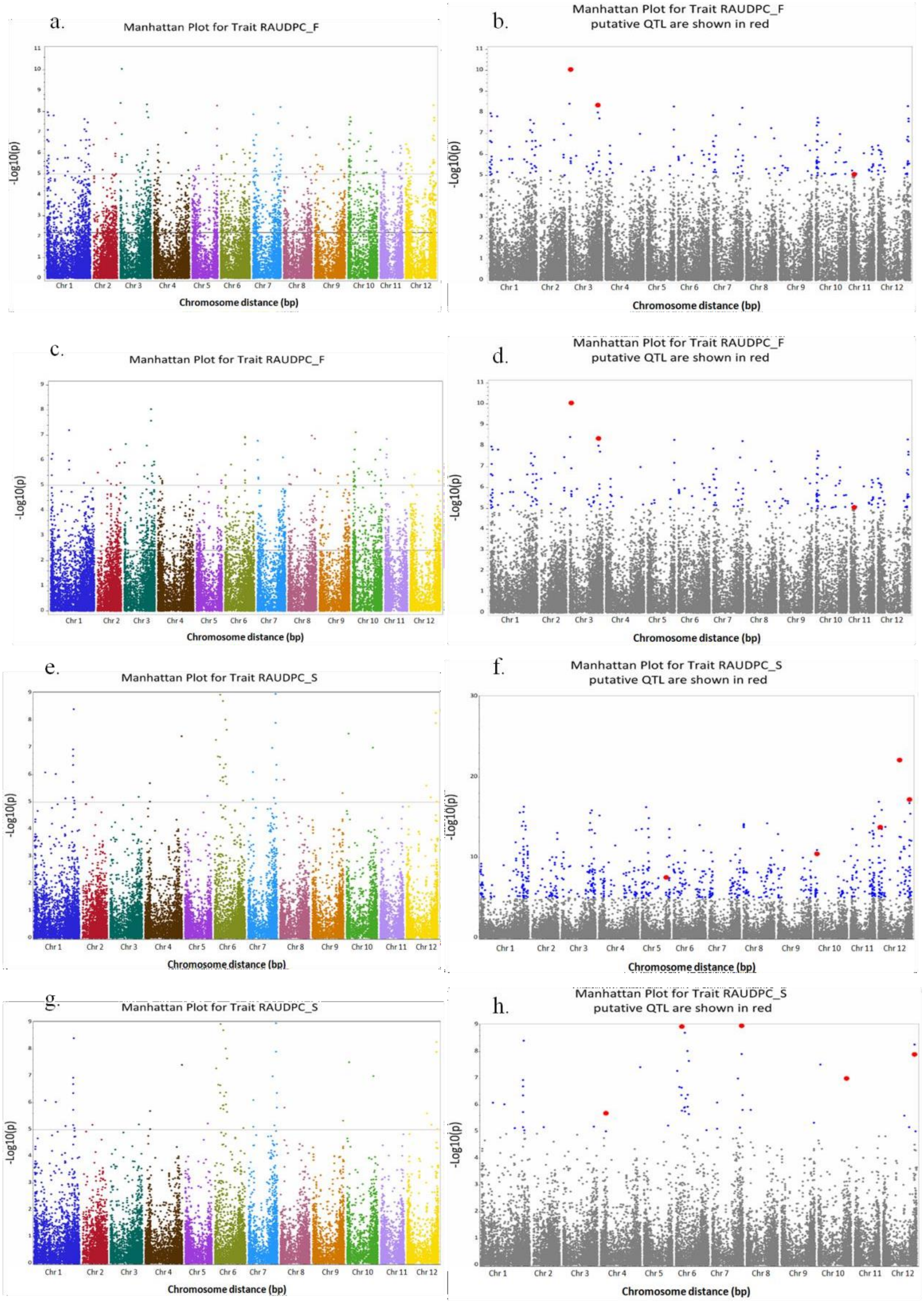

S3 Fig. Manhattan plots and the most informative SNP (putative QTL) for genome wide association study (GWAS) of resistance to *Phytophthora infestans* in Group Phureja. On the x-axis, different color tones correspond to different chromosomes within the genome from 1 to 12. The dashed horizontal line indicates the significant threshold at-log 10 (p-value) = 8. a.- d. Manhattan plot and putative QTL for resistance to *P.infestans* in foliage. a-b Correspond to SNP associated in La Union environment, c-d Correspond to SNP associated in Subachoque environment. e.-h. Manhattan plot for resistance to *P.infestans* in stem. e-f Correspond to SNP associated in La Union environment, g-h. Correspond to SNP associated in Subachoque environment.
